# Pancreatic Atrophy: A Narrative Review and Surgical Interpretation

**DOI:** 10.1002/ags3.70230

**Published:** 2026-05-07

**Authors:** Rika Fujino, Ken‐ichi Okada, Yohei Masugi, Eisuke Iwasaki, Masaki Mori

**Affiliations:** ^1^ Department of Gastroenterological Surgery Tokai University School of Medicine Isehara Japan; ^2^ Department of Pathology Tokai University School of Medicine Isehara Japan; ^3^ Division of Gastroenterology, Department of Internal Medicine Tokai University School of Medicine Isehara Japan

**Keywords:** focal, pancreatic atrophy, pancreatic ductal adenocarcinoma, pancreatic parenchyma, upstream

## Abstract

Despite recent advances in multimodal management, pancreatic ductal adenocarcinoma remains a fatal malignancy. Early detection of indirect findings of pancreatic ductal adenocarcinoma is essential to improve treatment outcomes, drawing attention to pancreatic parenchymal atrophy. Pancreatic parenchymal atrophy, defined as the narrowing of the parenchyma below a line connecting the cephalic and caudal margins of the lesion on computed tomography, can predict early‐stage pancreatic ductal adenocarcinoma and its intraductal extension. Computed tomography with three‐directional imaging is the ideal initial modality for diagnosing pancreatic parenchymal atrophy. In patients with early pancreatic ductal adenocarcinoma, pancreatic parenchymal atrophy exhibits a significantly longer intraductal lateral tumor extension. Pancreatic parenchymal atrophy can be resected using an appropriate‐margin pancreatectomy with a low risk of positive surgical margins. However, the appropriate surgical margin length for each case remains unclear. Carcinoma in situ may be present only within a focal pancreatic parenchymal atrophy region, and pancreatic duct changes may not be present. For such patients, a surgical strategy of limited pancreatectomy with a smaller surgical margin, combined with additional intraoperative resection based on frozen‐section results, is acceptable. Although the relationship between carcinoma in situ and fatty replacement of the pancreatic parenchyma has been investigated, the underlying mechanism remains unclear.

## Graphical abstract

Pancreatic parenchymal atrophy pattern and intraoperative margin assessment guide surgical strategy.

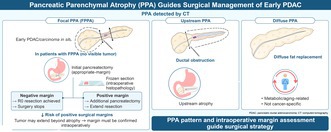

## 1 Introduction

**FIGURE 1:**
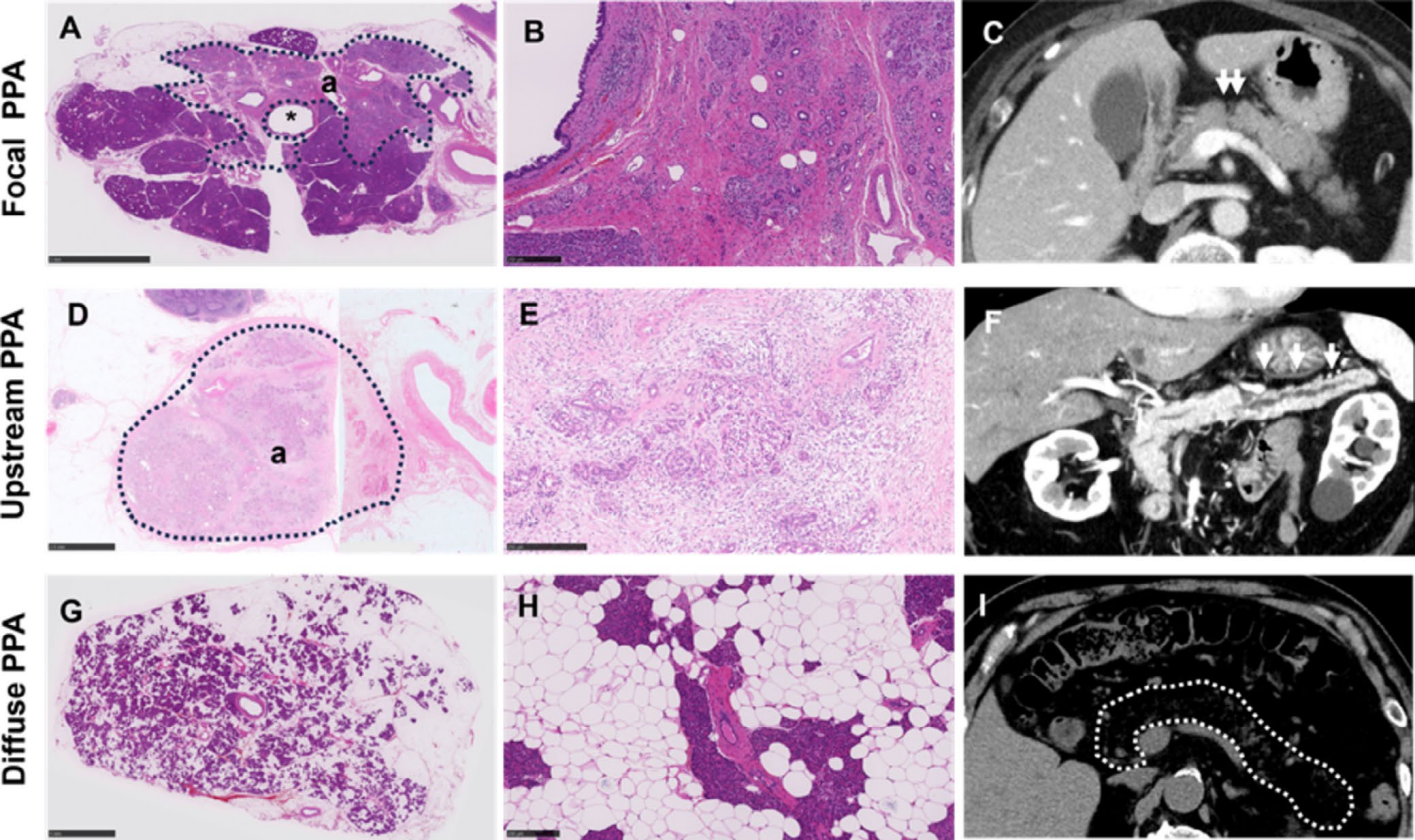
Types of pancreatic parenchymal atrophy (PPA). (A, B) Focal segmental PPA associated with high‐grade pancreatic intraepithelial neoplasia (A, loupe image; B, high‐magnification image). Neoplastic pancreatic ducts, including the main pancreatic duct (asterisk), are mildly dilated. The atrophic area (a) is outlined with a dashed line. (C) Contrast‐enhanced computed tomography (axial image) demonstrating focal segmental PPA corresponding to the pathological findings in (A) and (B). (D, E) Focal global PPA due to upstream obstruction of the main pancreatic duct (D, loupe image; E, high‐magnification image). (F) Contrast‐enhanced computed tomography (coronal image) demonstrating focal global PPA corresponding to the pathological findings in (D) and (E). (G, H) Diffuse PPA associated with marked fat replacement (G, loupe image; H, high‐magnification image). (I) Contrast‐enhanced computed tomography (axial image) demonstrating diffuse PPA corresponding to the pathological findings in (G) and (H). Dashed line indicates the pancreas. Bar = 5 mm (A, G), 2.5 mm (D), and 250 μm (B, E, H).

Pancreatic ductal adenocarcinoma (PDAC), a fatal malignancy, is expected to become the second leading cause of cancer‐related death in the United States by 2030 and is currently the fourth and seventh leading cause of cancer‐related deaths in Japan and industrialized countries, respectively [1, 2, 3, 4]. According to GLOBOCAN 2018 estimates, pancreatic cancer is the 11th most common cancer worldwide, with 458,918 new cases and 432,242 mortalities in 2018 [1]. The early detection of indirect findings of pancreatic cancer is essential to improve treatment outcomes, attracting attention to pancreatic parenchymal atrophy (PPA). In clinical practice, surgical resection is performed for PPA with main pancreatic duct stenosis, even when obvious pancreatic tumors are absent. PPA can develop through various mechanisms, including oxidative stress, toxic metabolism, pancreatic duct obstruction, pancreatic ischemic/necrotic fibrosis, pancreatitis‐induced fibrosis, fat replacement, and postoperative factors [5, 6, 7, 8, 9, 10]. PPA, defined as the narrowing of the parenchyma below a line connecting the cephalic and/or caudal margins of the lesion on computed tomography (CT) [11] (Figure 1), is a known predictor of early‐stage pancreatic cancer and intraductal extension of early PDAC [9, 12, 13, 14, 15, 16, 17]. Several studies on PPA form the basis for developing surgical strategies regarding the timing and extent of lesion removal and the histopathological diagnosis of the pancreatic resection margin. Patients with early‐stage pancreatic cancer or carcinoma in situ generally undergo limited pancreatectomy. However, decisions regarding surgical methods that allow sufficient pancreatic parenchyma removal without risking pathologic residual cancer are essential. Accordingly, the anatomical location and length of the pancreatic atrophy are crucial for determining the surgical method. Recent findings on indirect imaging of early‐stage pancreatic cancer/premalignant lesions are beginning to clarify the frequency and modality of imaging assessments for the follow‐up of high‐risk individuals or the remnant pancreas of patients who underwent pancreatic resection, including those with PPA [5, 18, 19, 20, 21, 22, 23]. Herein, we summarize how PPA predicts the intraductal extension of early PDAC, surgical management, and prognosis, aiming to enhance the identification, assessment, and management of patients with PPA.

## 2 Mechanisms/Significance of Pancreatic Atrophy and Its Association With Intra‐Pancreatic Fat Deposition

PPA is caused by several mechanisms. Free radicals generated by alcohol damage acinar cells, leading to inflammation and fibrosis; alcohol and its metabolites also directly damage acinar cells, leading to lipid accumulation and cell loss [6]. Additionally, alcohol and other substances increase protein concentrations in the pancreatic juice, which can form plugs that obstruct ducts. Acute pancreatitis causes acinar cell necrosis, leading to inflammation and fibrosis [6]. In an animal model, both the obstruction of pancreatic duct flow and parenchymal ischemia contributed to pancreatic atrophy, and impaired blood flow in the pancreatic parenchyma caused pancreatic fat replacement [7]. Pancreatic intraepithelial neoplasia (PanIN) can obstruct pancreatic juice flow, leading to parenchymal fibrosis and atrophy [17, 18]. Microscopically, pancreatic parenchymal fibrosis develops due to several factors, including pancreatic intraepithelial neoplasms (PanINs), dysplasia, hyperplasia, and chronic pancreatitis [5]. Additionally, pancreatic resection, stenosis, malnutrition, and deregulation of pancreatic neurohormonal stimulator mechanisms contribute to atrophy [5, 6, 7, 23, 24]. Importantly, pancreatic atrophy progression can result from the development and proliferation of malignant tumors [13, 16, 18].

PPA, characterized by the gradual replacement of normal pancreatic tissue with fat cells and fibroblasts, is associated with metabolic dysfunction and steatotic liver disease [25]. Conversely, sufficient evidence allows conceptualization of intrapancreatic fat deposition as a combination of intrapancreatic fat accumulation and pancreatic fat replacement [26]. Activation of the intracellular transcription factor peroxisome proliferator‐activated receptor γ (PPARγ) is considered to be involved in the mechanism. One study posits that PPARγ is activated in response to the progression of cancer localized within the epithelium, and surrounding adipogenesis may occur owing to PPARγ activation [27, 28, 29]. However, no theory regarding the relationship between carcinoma in situ and fatty replacement of the surrounding pancreatic parenchyma has been established.

## 3 Definition and Types of Pancreatic Parenchymal Atrophy

Pancreatic atrophy can arise from different etiologies with distinct distribution patterns and clinical implications: (i) tumor‐associated focal/segmental atrophy, (ii) upstream (tail‐side) atrophy secondary to main pancreatic duct stricture/obstruction, and (iii) diffuse atrophy related to metabolic factors or chronic inflammation. We stratified these into (i) focal PPA (FPPA), (ii) upstream PPA (UPPA), and (iii) diffuse PPA (DPPA). Morphologically, FPPA and UPPA were defined as narrowing of the parenchyma below a line connecting the cephalic and/or caudal margins of the lesion on CT [11] (Figure 1). FPPA presents as focal/segmental atrophy of the parenchyma adjacent to or surrounding the duct stenosis, or occurring without duct stenosis. UPPA presents as global atrophy that occurs caudal to the site of pancreatic duct stenosis [12, 18]. In contrast, a distinct type, DPPA, was defined as moderate‐to‐severe diffuse replacement of the whole pancreatic parenchyma by fat tissue on CT (Figure 1). In this review, we included all types of PPA to organize and clarify the etiologic classification, but excluded DPPA to focus on the investigation of surgical procedures. FPPA—which is crucial in pancreatic cancer—is often confused with overall pancreatic atrophy that occurs after pancreatitis or with pancreatic steatosis. Although FPPA is the main focus in the diagnosis of early pancreatic cancer, overall pancreatic atrophy becomes important in cases in which cancer arises from chronic pancreatitis. However, when it occurs after pancreatitis or with pancreatic steatosis, distinguishing between these conditions is difficult. In such cases, clinicians may establish the diagnosis by integrating findings from various types of multimodal imaging, focusing on the distribution of fat density/fibrotic changes in the pancreatic parenchyma, referring to changes in imaging findings over time, and reviewing the patient's past medical history.

## 4 Histopathology of Pancreatic Atrophy and Pancreatic Fat Replacement

**FIGURE 2:**
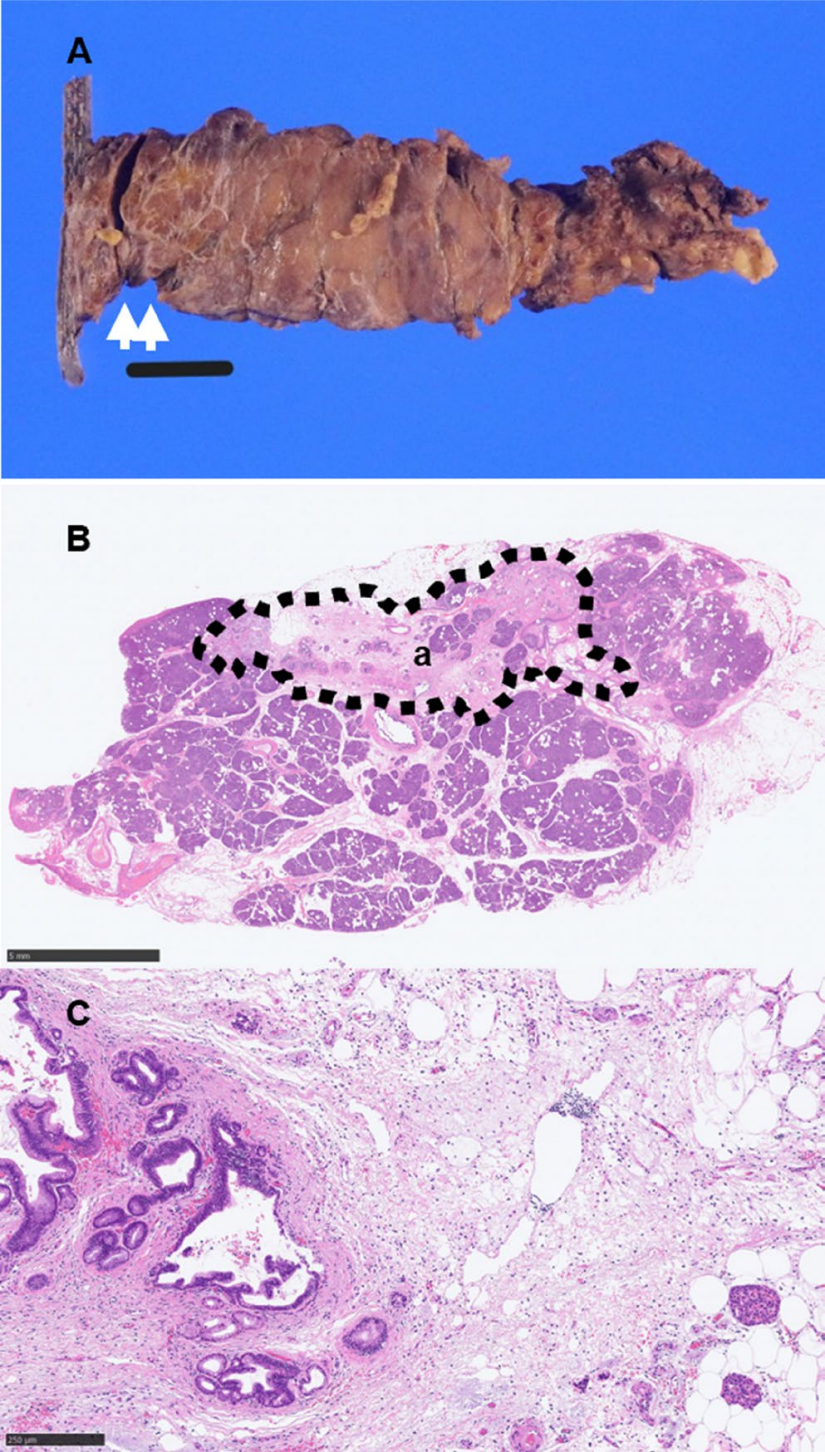
A case of focal pancreatic parenchymal atrophy (FPPA) associated with pancreatic intraepithelial neoplasia (PanIN). (A) Macroscopic image of the resected pancreas demonstrating focal atrophy of the pancreatic parenchyma in the pancreatic body (arrows). (B) Loupe image of FPPA associated with PanIN. The atrophic area (a) is outlined with a dashed line. (C) High‐magnification image of FPPA associated with PanIN. Bar = 1 cm (A), 5 mm (B), and 250 μm (C).

PPA—a dynamic process caused by an imbalance between regeneration and loss of pancreatic epithelial cells, including acinar and ductal cells—is characterized by a significant loss of pancreatic acinar cells, which typically comprise > 90% of the pancreatic parenchyma. Histopathologically, in PPA, the pancreatic parenchyma is replaced by adipocytes. Therefore, pancreatic atrophy and fat replacement are considered two sides of the same coin. In literature, pancreatic fat replacement is also referred to as pancreatic fatty replacement, pancreatic lipomatosis, fat infiltration, pancreatic fatty change, fat pancreas, lipomatous pseudohypertrophy, and pancreatic steatosis in the description of histopathology [30, 31]. However, no consensus exists regarding the definition and diagnostic criteria for pancreatic fat replacement. Pathologically, pancreatic fat replacement has been defined as replacement of > 25% of the pancreatic parenchyma by adipocytes [32]. Conversely, several recent reports have suggested the definition of replacement of > 10% of the parenchyma by fatty tissue [33]. Regardless of the degree of pancreatic fat replacement, the lobular structure of the pancreatic parenchyma generally remains well‐preserved. In cases of severe fat replacement, the boundary with the surrounding retroperitoneal fat becomes indistinct, resulting in microscopic findings where only the pancreatic ducts or islets remain within the adipose tissues (Figure 2).

**FIGURE 3:**
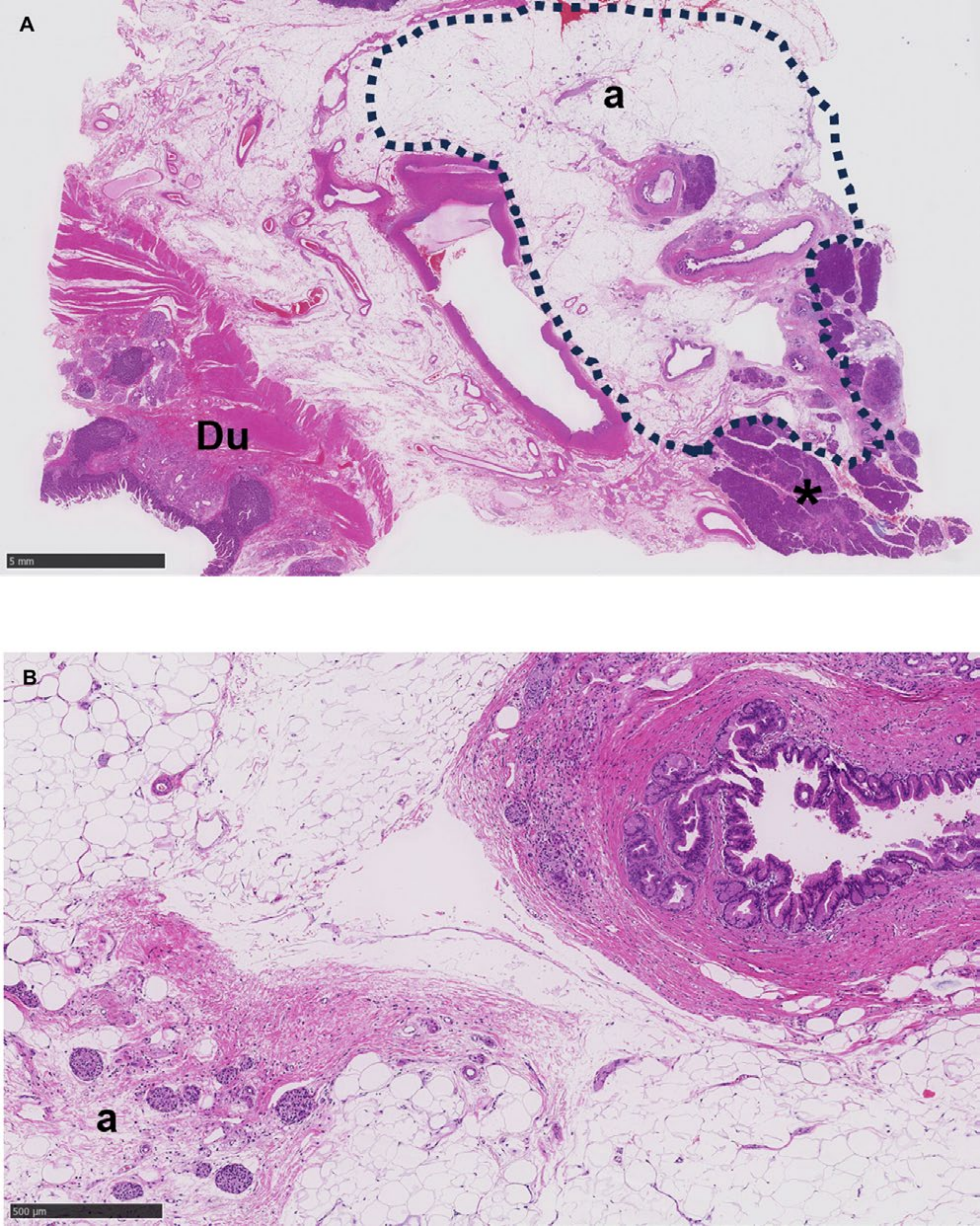
Focal segmental pancreatic parenchymal atrophy (PPA) in the groove area associated with intraductal papillary mucinous neoplasm (IPMN). (A) Loupe image of focal segmental PPA associated with IPMN. The atrophic area (a), outlined with a dashed line, is located in the groove area (Du: Duodenum). No apparent atrophy is observed in the adjacent pancreatic parenchyma without IPMN (asterisk). (B) High‐magnification image of the atrophic area. Residual islet cells are seen in the severely atrophic area (a). Bar = 5 mm (A) and 500 μm (B).

Pancreatic fat replacement is a common pancreatic condition, with an estimated global prevalence of at least 16% of the population [30], most of whom are asymptomatic. The average fat content of the pancreas of individuals in their 80s was reportedly 35%, indicating that its prevalence increases with age [34]. Magnetic resonance imaging (MRI) findings of pancreatic atrophy—lobulation and fatty degeneration—are characteristic age‐related changes. It is necessary to recognize these changes when interpreting abdominal MRI findings in patients with and without pancreatic disease [35]. Pancreatic fat replacement can be diffuse or localized. Diffuse pancreatic fat replacement has been linked to various pancreatic and systemic diseases, including acute pancreatitis, chronic pancreatitis, pancreatic neoplasms, diabetes, and arteriosclerosis. Shwachman–Diamond syndrome, a rare genetic disorder that causes primary lipomatous pseudohypertrophy, is characterized by aplastic anemia and pancreatic exocrine dysfunction [36]. Conversely, localized fat replacement is frequently associated with PanIN and intraductal papillary mucinous neoplasms (IPMN) (Figure 3) [37, 38, 39]. Recently, localized pancreatic atrophy or localized pancreatic fat replacement has gained attention because imaging findings related to early pancreatic cancers may not manifest as tumor masses. High‐grade PanIN is recognized macroscopically not by the presence of a mass or significantly dilated ducts but by an indirect finding of localized fat replacement [40]. Thus, localized pancreatic fat replacement can prompt further histopathological examinations, such as pancreatic duct cytology, endoscopic ultrasound (EUS)‐guided pancreatic biopsy, or EUS‐guided fine‐needle aspiration.

## 5 Detection of Pancreatic Atrophy and Further Evaluation for Surgical Procedures

In a recent large‐scale study, suspicious PDAC findings on prediagnostic CT were observed in 47.8% of patients with PDAC, with the most characteristic finding being progressive FPPA approximately 2.7 years pre‐diagnosis, followed by progressive main pancreatic duct dilatation approximately 1.1 years pre‐diagnosis. In PDAC, FPPA more frequently occurs in the pancreatic body/tail than in the head [19]. Focal pancreatic steatosis observed on CT may be an indicator of pancreatic carcinoma in situ (PCIS) [12, 19, 41, 42, 43, 44]. Therefore, the ideal initial diagnostic modality for PPA is CT with three‐directional imaging. In a systematic review, the pooled prevalence of intrapancreatic fat deposition in individuals with pancreatic cancer or premalignant lesions was 52% [25]. The presence of pancreatic cancer or premalignant lesions was associated with a significantly increased risk of intra‐pancreatic fat deposition [26]. Accordingly, MRI/magnetic resonance cholangiopancreatography and EUS of intrapancreatic fat deposition serve as sensitive tools for evaluating PPA lesion distribution [21, 35, 45, 46, 47, 48, 49]. EUS provides sufficient resolution to observe pancreatic changes in PCIS, which are often accompanied by chronic pancreatitis and fatty infiltration in the pancreatic background [47, 48, 50]. Additionally, we previously reported that intraductal ultrasonography is useful for evaluating intraductal epithelial tumor distribution as it allows simultaneous observation of intraductal lesions and peripancreatic anatomy to guide resection [51]. Finally, cytological evaluation of the main pancreatic duct by endoscopic retrograde cholangiopancreatography (ERCP) using serial pancreatic juice aspiration cytological examination is essential for diagnosing the presence of cancer cells [52, 53, 54, 55, 56]. However, because the sensitivity of pancreatic fluid cytology using endoscopic nasopancreatic drainage for pancreatic cancer diagnosis is not particularly high, and concerns remain regarding post‐ERCP pancreatitis risk, this approach should be considered only in selected patients with PPA without detectable tumors, and only if the results are expected to influence subsequent treatment, with careful consideration of the risks and benefits. Furthermore, endoscopic ultrasound‐guided fine‐needle aspiration, although its diagnostic performance is highly operator‐dependent, may be recommended for pathological diagnosis when a tumor is identified.

## 6 The Association Between Pancreatic Atrophy Pattern and Intraductal Cancer Extension

Few studies have investigated the optimal surgical procedures and PPA location and distribution. However, one study proposed two distinct types of PPA—FPPA and UPPA—indicating the presence and distribution of early pancreatic cancer [11]. Therein, cases with FPPA or UPPA showed significantly longer intraductal lateral cancer extensions than those without atrophy (median: 20.0 vs. 5.0 mm); two patients with FPPA or UPPA showed positive resection margins despite wide pancreatic resection for atrophic areas, and three patients showed recurrence in the remnant pancreas. Thus, even if the intraductal carcinoma extension has spread more than twice the PPA length, securing an appropriate surgical margin can significantly reduce the positive margin rate [11]. Although total pancreatectomy should be avoided when feasible, additional pancreatectomy should be performed based on the histopathological diagnosis of frozen sections, not only for positive surgical margins of appropriate length but also in areas with chronic pancreatitis or pancreatic fat infiltration [19, 26].

## 7 Optimal Surgical Procedures Based on Pancreatic Atrophy Pattern and Location

In PDAC, PPA is more likely to occur in the pancreatic body/tail than in the head [19]; thus, distal pancreatectomy is the most common surgical procedure. As FPPA and UPPA show longer cancer extensions beyond the pancreatic atrophy area, determining the extent of pancreatic parenchymal resection along the main pancreatic duct is challenging, especially in patients with pancreatic body lesions. Therefore, positive resection margins are more likely with limited pancreatectomy—such as a middle pancreatectomy—for the removal of only FPPA areas. Additionally, determining the transection site with an appropriate margin from the edge of the atrophic lesion and choosing pancreatoduodenectomy or distal pancreatectomy based on the anatomical location of the lesion is preferable [11, 51]. Collectively, FPPA or UPPA in the pancreatic head or tail can be resected by an appropriate margin pancreatoduodenectomy or distal pancreatectomy, respectively, with a low risk of positive surgical margins. For FPPA in the pancreatic body, pancreatoduodenectomy with an appropriate margin removing the pancreatic head/body for additional intraoperative resection is preferred owing to its technical ease. In distal pancreatectomy, by lifting the gastroduodenal artery after branch ligation and taping, pancreatic resection towards the duodenal side up to approximately 20–30 mm is possible from the midline in front of the portal vein. If additional resection nears the pancreatobiliary junction, prompt conversion to total pancreatectomy is necessary to avoid injury to the bile duct or duct of Santorini. Indications for several specialized surgeries, including duodenum‐preserving total pancreatic head resection or removal of the uncinate process, were not considered herein due to a lack of evidence regarding surgical outcomes for PPA. However, PCIS may be present within the FPPA region alone, and pancreatic duct changes may not be evident. For such patients, a surgical strategy of limited pancreatectomy with a smaller surgical margin, combined with additional intraoperative resection based on frozen‐section results, is also acceptable. In the current era, with limited data on FPPA, the appropriate surgical margin length for each case remains unclear. Therefore, surgeons should decide on the initial transection line and the extent of additional intraoperative resection while considering both the risk of residual tumor and the function of the remnant pancreas.

## 8 Surgical Strategies for Pancreatic Atrophy With/Without Detectable Tumors

In a previous Japanese multicenter study of early pancreatic cancer, stage 0 pancreatic tumors were seldom detectable using imaging modalities [56, 57]. However, if a tumor is detectable preoperatively using any modality, determining whether the final tumor diameter is greater or less than 10 mm may be histologically challenging, potentially resulting in a difference in clinical prognosis [13]. According to the Clinical Practice Guidelines for Pancreatic Cancer 2025 from the Japan Pancreas Society, the treatment algorithm for stage I pancreatic cancer recommends standard surgery and systemic treatment, as even T1 PDAC demonstrates aggressive behavior [58, 59, 60]. Therefore, if T1 PDAC is suspected, regional lymph node dissection should be performed based on the lesion location. Although no randomized controlled trials have compared limited pancreatic resection with standard pancreatic resection techniques—including pancreaticoduodenectomy or distal pancreatectomy—in terms of postoperative prognosis after resection of early pancreatic cancer, middle pancreatectomy is considered preferable only for patients preoperatively diagnosed with PCIS without a detectable tumor, regardless of the presence of main pancreatic duct stenosis [56].

## 9 Repeated Pancreatectomy for the Remnant Pancreas

PCIS may recur as residual pancreatic cancer after resection in cases with incomplete pancreatic atrophy harboring residual PCIS, or it may arise at a separate location [23, 24]. Previous studies have reported that a positive pancreatic transection margin is a risk factor for remnant pancreatic recurrence, and repeated pancreatectomy is recommended for patients without extrapancreatic recurrence. All patients with PCIS should undergo continual surveillance every 6 months for > 5 years after surgery to monitor for remnant pancreatic recurrence and may be considered for repeated pancreatectomy if recurrence is detected. If it is technically difficult to preserve the pancreas, total remnant pancreatectomy should be considered in carefully selected patients, taking into account the patient's age and systemic condition.

## 10 Spleen‐Preserving Distal Pancreatectomy (SPDP) for Pancreatic Body/Tail Atrophy

SPDP is usually indicated for borderline malignant and clear boundary tumors, such as IPMN, mucinous cystic neoplasm, solid pseudopapillary neoplasm, serous cyst neoplasm, pancreatic neuroendocrine neoplasm, or metastatic pancreatic tumor of renal cell carcinoma. Clinically, if a pancreatic body/tail tumor is preoperatively diagnosed as growing within the pancreatic duct or free from splenic vessels, SPDP should be considered, given its benefits [61]. Thus, PPA and main pancreatic duct stenosis without any detectable mass on imaging are considered good indications for SPDP, since these lesions are mostly associated with PanIN, IPMN, or PCIS. In recent years, minimally invasive surgeries have improved the feasibility and safety of SPDP [62]. SPDP for PPA without detectable tumors using a minimally invasive approach is expected to increase the incidence of curative resection of early‐stage PDAC, leading to improved overall surgical outcomes for all‐stage PDAC. However, middle pancreatectomy or SPDP without lymph node dissection should be avoided for clinical T1 PDAC, as even tumors < 10 mm may exhibit regional lymph node metastasis or histopathological invasion into the periarterial neural plexuses of the splenic artery or venous walls, depending on their location in the pancreatic parenchyma [63, 64].

## 11 Prognosis and Follow‐Up for Remnant Pancreas After PPA Resection

Usual postoperative surveillance for PPA should be implemented for patients with pathologically confirmed PDAC. A previous study reported postoperative recurrence in the remnant pancreas even in patients with early‐stage PDAC who had a negative final surgical resection margin [11]. Patients with microscopic residual epithelial tumors in the pancreatic duct on histopathological examination should be carefully followed, considering early recurrence in the remnant pancreas [11]. Furthermore, patients with PPA accompanied by fat deposition in the parenchyma exhibit increased potential to develop additional PPA in the future [26]. The European Society for Medical Oncology recommends against routine surveillance and periodic additional examinations, based on poor prognosis at the time of recurrence and the limited availability of effective treatments. Nevertheless, for patients with elevated serum carbohydrate antigen (CA) 19‐9 levels at diagnosis, French guidelines recommend monitoring for early recurrence by measuring serum CA19‐9 levels and performing chest, abdominal, and pelvic CT every 3 months for 2–3 years, followed by every 6–12 months for up to 5 years. Both the National Comprehensive Cancer Network and the Japan Pancreas Society recommend routine testing for serum CA19‐9 and abdominal and pelvic CT imaging every 3–6 months for the first 2 years post‐surgery [65, 66].

Absence of perineural invasion and a low tumor grade are associated with long‐term (10‐year) survival in patients with PDAC [60]. Tumor size is the strongest predictor of survival in patients with early PDAC. In patients with PCIS, the predicted 5‐year survival rates associated with the final pathological T stage are as follows: for PCIS, an overall survival (OS) of 76% and a cancer‐specific survival of 84%; for tumors smaller than 1 cm, an OS of 36%, and a cancer‐specific survival of 40%; and for tumors measuring 1.1–2.0 cm, an OS of 17% and a cancer‐specific survival of 20%, respectively [60].

## 12 Discussion

Recent studies have highlighted the role of PPA in early‐stage pancreatic cancer. Currently, in clinical practice, no standard surgical treatment exists for PPA in cases with stenosis of the main pancreatic duct recognized on imaging but without a detectable tumor mass. Conversely, recent studies revealed that most clinicians proceed with surgery for patients with suspected high‐grade PanIN or PCIS presenting with PPA and stenosis of the main pancreatic duct without any evident malignant cells. However, the evidence is insufficient to verify the relationship between imaging findings—including main pancreatic duct stenosis and pancreatic atrophy—and pathological findings—including spatial distribution and oncological characteristics of the tumor.

As surgical outcome assessments for PPA are limited, herein, we developed a fundamental surgical strategy by investigating available studies on this topic. Distal pancreatectomy is the most common surgical procedure, as it is more likely to be performed for PDAC in the pancreatic body/tail than for PDAC in the pancreatic head. PPA in the pancreatic head or tail can be resected using an appropriate‐margin pancreatoduodenectomy or distal pancreatectomy, respectively, with a low risk of positive surgical margins. For PPA in the pancreatic body, an appropriate‐margin pancreatoduodenectomy removing the pancreatic head/body is preferred, as it allows for easier additional resection. However, the optimal length of the surgical margin for each case remains unclear. Therefore, surgeons should determine the initial transection line and the extent of additional intraoperative resection while considering the risk of residual tumor and function of remnant pancreas. Although total pancreatectomy should be avoided when feasible, additional pancreatectomy of an appropriate length should be performed based on the histopathological diagnosis of frozen sections to exclude positive surgical margins, including the presence of high‐grade PanIN. Because evidence regarding prognosis after curative PPA resection is limited, the usual follow‐up protocol should be implemented according to the PDAC guidelines. The remnant pancreas after curative PPA resection may still develop additional PPA due to histological changes in the parenchyma with fat deposition [26].

Over the last decade, studies have addressed some of these issues; however, our understanding of PPA and PDAC remains limited. In multimodal imaging, PPA exhibits several histological changes, including focal narrowing of the parenchyma, fat replacement, and pancreatic duct stenosis with upstream pancreatic atrophy. Most studies on PPA in patients with PDAC are retrospective; therefore, they defined PPA using imaging techniques, especially CT. Future studies should focus on new findings regarding the association between epithelial changes in the pancreatic duct and pancreatic carcinogenesis from the perspective of molecular biology in patients with PPA, fat replacement, or premalignant changes [67, 68, 69, 70]. The direct effect of PPA on short‐ and long‐term outcomes in patients with PDAC remains unclear. Currently, no study has determined whether PPA itself results from cancer or is linked to carcinogenesis in PDAC. Nevertheless, several studies have demonstrated an association between PPA and early‐stage pancreatic cancer. However, the wide range of conditions, from PCIS to T1 PDAC, poses significant clinical challenges. T1 PDAC, despite its small size, exhibits aggressive behavior, warranting active local surgery and systemic chemotherapy. Therefore, further examination for the presence of detectable tumors is essential to determine surgical treatment after finding PPA. Even in cases with no detectable tumor or stenosis of the main pancreatic duct, patients with PPA should be followed up using multimodal imaging and cytological assessments, with the intention of surgery within the status of PanIN or PCIS. Herein, we demonstrated that PPA location at the time of diagnosis of early‐stage PDAC and intraductal cancer extension may be significant factors in surgical planning. However, evidence regarding the association between the length of the intraductal cancer extension and PPA treatment is scarce, warranting further nationwide studies.

## Author Contributions

**Eisuke Iwasaki:** writing – review and editing, investigation. **Yohei Masugi:** investigation, writing – review and editing. **Rika Fujino:** writing – original draft. **Masaki Mori:** writing – review and editing. **Ken‐ichi Okada:** writing – original draft.

## Funding

The authors have nothing to report.

## Ethics Statement

The authors have nothing to report.

## Consent

The authors have nothing to report.

## Conflicts of Interest

Dr. Masaki Mori serves as an Editorial Board Member of *Annals of Gastroenterological Surgery*. The other authors declare no conflicts of interest.

## Data Availability

The authors have nothing to report.
